# Influence of silica nanoparticle incorporation on the wettability, oil affinity, and dye removal properties of blow-spun polymeric membranes

**DOI:** 10.1007/s10853-026-13279-6

**Published:** 2026-07-15

**Authors:** A. S. Bento, M. Brito, A. Rovisco, A. C. Baptista, I. Ferreira

**Affiliations:** CENIMAT|i3N, Department of Materials Science, School of Science and Technology, NOVA University Lisbon, Caparica, Portugal

## Abstract

The versatility and scalability of blow spinning motivated us to investigate the surface properties of blow-spun poly(ε-caprolactone) (PCL), cellulose acetate (CA), polystyrene (PS), and their blends, with and without silica nanoparticles (SiO_2_ NPs). Pristine PCL and PS membranes are hydrophobic and oleophilic, whereas CA membranes are hydrophilic and oleophilic. Incorporation of SiO_2_ NPs renders PCL membranes superhydrophilicity while preserving oleophilicity. Blending PS with CA or PCL yields hydrophobic and oleophilic membranes. These characteristics enable selective oil uptake and either water repellence or water spreading, depending on formulation. Dye removal was assessed using methylene blue (MB), showing rapid uptake on PS/CA and higher overall removal for PCL–SiO_2_ NPs membranes. SiO_2_ NPs incorporation was confirmed by Fourier transform infrared (FTIR) spectroscopy and scanning electron microscopy (SEM). Under the conditions tested (1 mL MB solution), a PCL-SiO_2_ NPs membrane (6.9 cm^2^, 118 μm thick, 24.6 mg) reduced the MB concentration by ~ 4 mg L^−1^ within 19 h (~ 4 μg removed; apparent uptake ~ 0.16 mg g^−1^). Overall, blow spinning provides an accessible route to obtain membranes for oil–water separation and dye retention.

**Graphical abstract:**

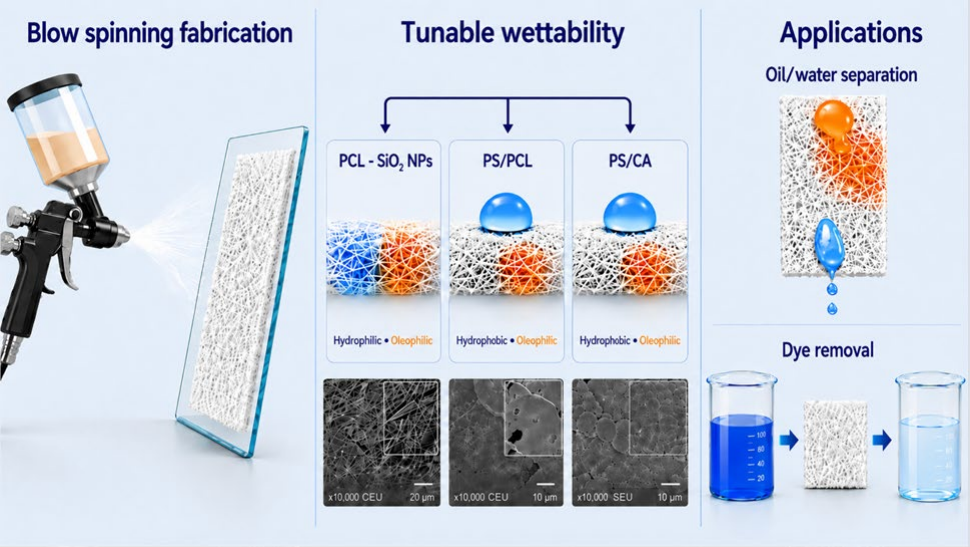

**Supplementary Information:**

The online version contains supplementary material available at 10.1007/s10853-026-13279-6 .

## Introduction

The effective, low-cost, and scalable fabrication of porous polymer membranes with controlled wettability remains important for oil–water separation and water treatment. Electrospinning is widely used to prepare fibrous membranes with high surface area, but its throughput is typically limited for large-area applications. Blow spinning is a simpler, higher-throughput alternative that can generate fibers and film–fiber hybrid morphologies and can be implemented as free-standing membranes or coatings on substrates.

Poly(ε-caprolactone) (PCL) is a biodegradable aliphatic polyester widely used in biomedical applications, including wound dressing, drug delivery, and tissue engineering scaffolds, due to its tunable degradation rate and biocompatibility [1]. As PCL is inherently hydrophobic, surface hydrophilization is often required to improve cell adhesion. Reported approaches include plasma treatment [2], hydrolysis and aminolysis (e.g., NaOH-based treatments) [3], blending with hydrophilic polymers [4], blending with PLGA [5], and incorporation of cartilage-derived extracellular matrix [6]. Beyond biomedical use, PCL-based composites have also been explored for adsorption applications, including removal of heavy metal ions from water [7].

Cellulose acetate (CA), obtained by acetylation of cellulose, is a renewable and widely used polymer in textiles, membranes, and filtration systems. Owing to polar functional groups in its structure, CA typically exhibits hydrophilic behavior. Nonetheless, hydrophilic-to-hydrophobic surface modifications have been reported for electrospun CA membranes, yielding super-water-repellent oil sorbents [8]. CA coatings produced by electrostatic spray deposition on stainless steel meshes and subsequently modified by chemical vapor deposition of methyltrichlorosilane (MTCS) have shown excellent oil/water separation performance, with reported superhydrophilicity and antifouling capability [9]. Additional strategies include chemical modification of cross-electrospun amidoximated polyacrylonitrile/regenerated cellulose acetate composites [10], sandwich Janus structures based on CA and polyurethane (PU) nanofibers [11], and CA/zeolite electrospun membranes [12]. Amphiphilic CA membrane incorporating oleophilic MoS_2_ nanoparticles have also been reported for oil-in-water separation with high water permeation and resistance [13].

Single-step electrospinning of polystyrene (PS) has been used to produce superhydrophobic-superoleophilic membranes on stainless steel meshes for selective oil absorption and efficient oil–water separation [14]. Aminated polystyrene-polymaleic(anhydride) (PSMM-NH_2_) hollow microspheres have been deposited on glass to form membranes for separating various oil–water mixtures [15]. Polystyrene–halloysite nanotube (PS-HNT) membranes produced by ultrasound-assisted solution casting method have been explored for water purification [16]. Electrospun PS membranes that were mechanically compressed have also been reported for efficient water-in-oil emulsion separation [17]. Hydrophobic microporous PS fibers produced by phase separation-assisted electrospinning have shown oil adsorption capability, with Ag-ZnO used to tune pore size and distribution [18]. Multilayer structures based on alternatively stacked PS-CA and PS/CA-SiO_2_ (or PS/CA-HSiO_2_) microfiber sub-membranes have been tested to improve oil adsorption performance [19]. Effective oil–water separation has also been demonstrated using blow-spun PLA/SiO_2_ nanofiber membranes [20].

In this work, we provide a comparative assessment of blow-spun membranes fabricated from PCL, CA, PS, and their blends (PS/CA and PS/PCL), produced with and without SiO_2_ nanoparticles. The novelty lies in using a single scalable deposition approach (blow spinning) to access distinct wetting states (hydrophobic, hydrophilic, amphiphilic, and superhydrophilic/oleophilic) across multiple polymers and blends, and to relate droplet adhesion/sliding behavior (tilt/rotation tests) to oil–water selectivity and MB uptake. Our results show that the same processing route can yield membranes optimized either for selective oil uptake (low water adhesion) or for rapid water spreading (superhydrophilicity), depending on polymer composition and nanoparticle incorporation.

## Experimental details

**Synthesis of silica nanoparticles—**Silica nanoparticles were synthesized following an adapted procedure from Li et al. [21]. CTAB (0.2 g) was dissolved in 96 mL deionized water, then NaOH (0.7 mL, 2 M) was added under stirring, and the mixture was heated to 80 °C. After thermal stabilization, TEOS (1.20 mL) was rapidly added under vigorous stirring. The solution turned milky within approximately 1 min, indicating nanoparticle formation. The suspension was dried by solvent evaporation. The precipitate was washed repeatedly with ethanol to remove CTAB and residual impurities and then dried.

**Polymer solutions—**Cellulose acetate (CA) was dissolved in acetone: N, N-dimethylacetamide (DMAC) 3:1 (v/v); polystyrene (PS) was dissolved in N, N-dimethylformamide (DMF) at 5% (w/v); and poly(ε-caprolactone) (PCL) was dissolved in dichloromethane (DCM):DMF (3:1 v/v). Solutions were stirred until homogeneous.

**Solutions with silica nanoparticles** – SiO_2_ NPs (200 mg) were dispersed in 3.0 mL of the corresponding solvent system by vigorous stirring until visually homogeneous. Subsequently, 8.0 mL of polymer solution was added, and mixture was stirred until homogeneous prior to deposition.

**Polymer blend solutions** – PS/CA: PS (1.25 g) was dissolved in DMF (6.25 mL). After complete dissolution, acetone (18.75 mL) was added, followed by CA (0.94 g) until fully dissolved (PS:CA mass ratio ≈ 1.33:1). PS/PCL: Two 5% (w/v) solutions (PS and PCL) were prepared independently in DCM and mixed 1:1 (v/v).

**Figure 1:**
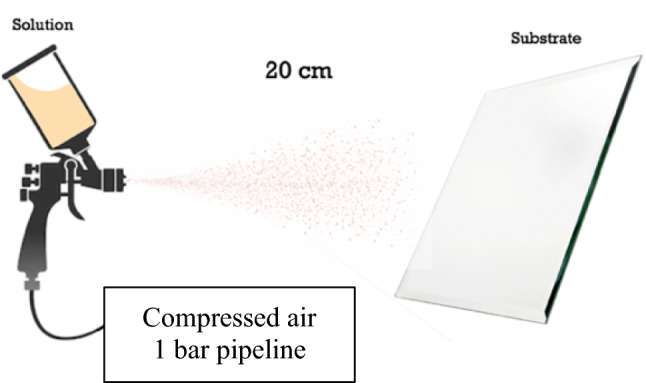
Schematic of blow spinning of polymers onto a metal or glass substrate.

**Production of membranes by blow spinning—**A compressed-air spray gun (1 bar) was used to atomize the polymer solution toward a glass substrate tilted at 45°. The nozzle–substrate distance was 20 cm as schematized in Fig. 1. After deposition, the samples were dried at room temperature until solvent evaporation was complete. Free-standing membranes were detached from the substrate for characterization.

**Characterization—**Optical microscopy (Leica DMi8) and scanning electron microscopy (SEM, Hitachi SU8220) were used to assess morphology. Raman (Witec Alpha 300 RAS, 532 nm) and Fourier transform infrared spectroscopy (FTIR, PerkinElmer Spectrum Two FTIR Spectrometer) spectra were collected for chemical identification. Thickness was measured using a handheld coating thickness gauge (Fischer Handheld Instrument Series FMP 10–20).

**Wettability characterization—**Static water contact angles (WCA) were measured with a DataPhysics OCA system at room temperature using sessile droplets (typical droplet volume 1 µL). For each membrane, contact angles were measured at multiple locations (minimum n = 3), and results are reported as average ± standard deviation. For very low contact angles (rapid spreading/absorption), the surface was classified as superhydrophilic, and the apparent angle is reported qualitatively (below the reliable measurement limit).

**Oil/water separation tests** – Oil–water selectivity was evaluated using two configurations: (i) a qualitative two-phase droplet test at 90° (Fig. 6) to visualize phase selectivity and droplet mobility and (ii) a quantitative gravity-driven collection test at 45° to estimate water recovery. For the droplet test, a combined oil–water droplet was deposited on the membrane surface and imaged during tilting/rotation. For water recovery measurements, a 1:1 (v/v) oil–water mixture (0.5 mL water and 0.5 mL oil) was dispensed perpendicularly onto the membrane (5 × 10 cm^2^), after which the membrane was tilted to 45° to allow water to drain and be collected, while the oil was retained within the membrane. Separation typically occurred within 2–3 min; the exact time depended on manual tilting, feeding conditions, and inclination. Water recovery was calculated as follows:

$$\text{Water Recovery (\%)}=\left(\frac{{V}_{\text{Water, collected}}}{{V}_{\text{Water, initial}}}\right)\times 100$$

where ${V}_{\text{Water, collected}}$ is the volume of water recovered after separation, and ${V}_{\text{Water, initial}}$ is the initial volume of water in the mixture.

**Dye uptake tests** – MB uptake was evaluated for PS/CA and PCL–SiO_2_ NPs by sequential immersion in an aqueous MB solution. Solution absorbance (calibration in Figure S1; example spectra in Figure S2–S3) and membrane reflectance (Figure S4) were monitored as function of cumulative immersion time. Absorbance spectra were recorded using a T90 + UV/VIS Spectrometer (PG Instruments Ltd.), and reflectance spectra were acquired using a JASCO V-770 UV–Vis/NIR spectrophotometer (NORLEQ).

## Results and discussion

**Figure 2:**
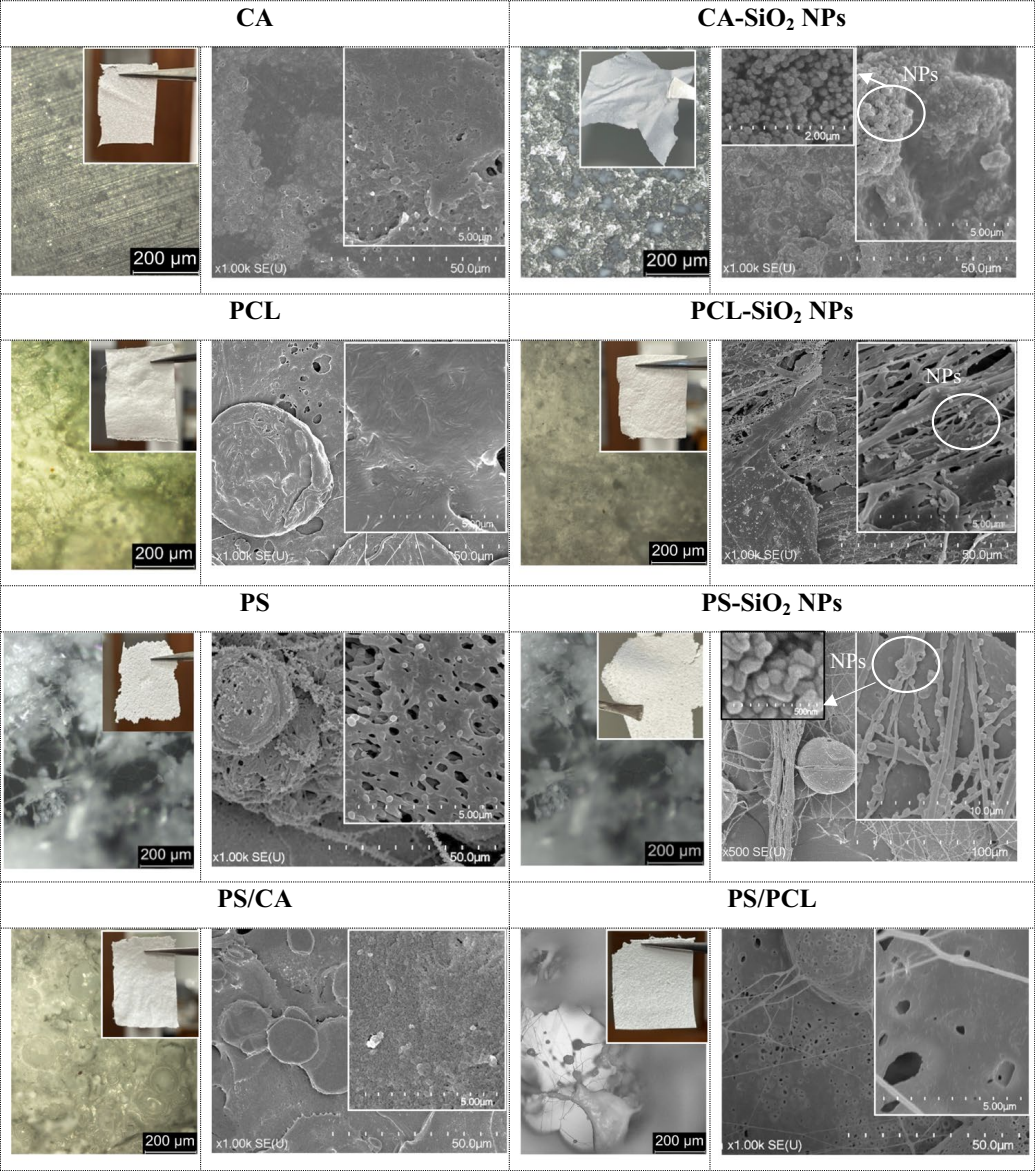
Photographs, microscopic images, and SEM images of the surface morphology of membranes.

The morphology of membranes obtained for each polymer, with and without SiO_2_ NPs, is shown in Fig. 2. The membranes were sprayed onto glass substrates and subsequently detached for characterization. Photographs, optical microscopy, and SEM reveal marked differences in texture and morphology between compositions. CA membranes (6.9 ± 1.3 μm thick) were flexible, paper-like, and tended to curl. When produced from CA solutions containing SiO_2_ NPs, the membranes (7.8 ± 1.2 μm) were less malleable and opaque. SEM images show a compact film containing SiO_2_ NPs agglomerates (< 200 nm) with individual nanoparticles with an average size of 120 ± 35 nm.

Pristine PCL membranes were flexible and elastic, with thickness of 22.7 ± 15.8 μm and 58.2 ± 27.4 μm for PCL-SiO_2_ NPs. SEM images indicated mixed fiber/film morphologies, suggesting that droplet deposition accompanied fiber formation. PCL-SiO_2_ NPs membranes appear more fibrous, and nanoparticles are visible.

PS membranes exhibit high porosity. With SiO_2_ incorporation, fibers were more clearly defined, and nanoparticle agglomerates were observed. Thickness is 117.7 ± 17.6 μm for PS and 141.3 ± 22.9 μm for PS–SiO_2_ NPs. Blends membranes (PS/CA and PS/PCL) show porosity influenced by PS. PS/CA (229.8 ± 108.4 μm) exhibited a more compact but less uniform morphology than PS/PCL (118 ± 20.21 μm), in which fibers and pores are more clearly observed.

**Figure 3:**
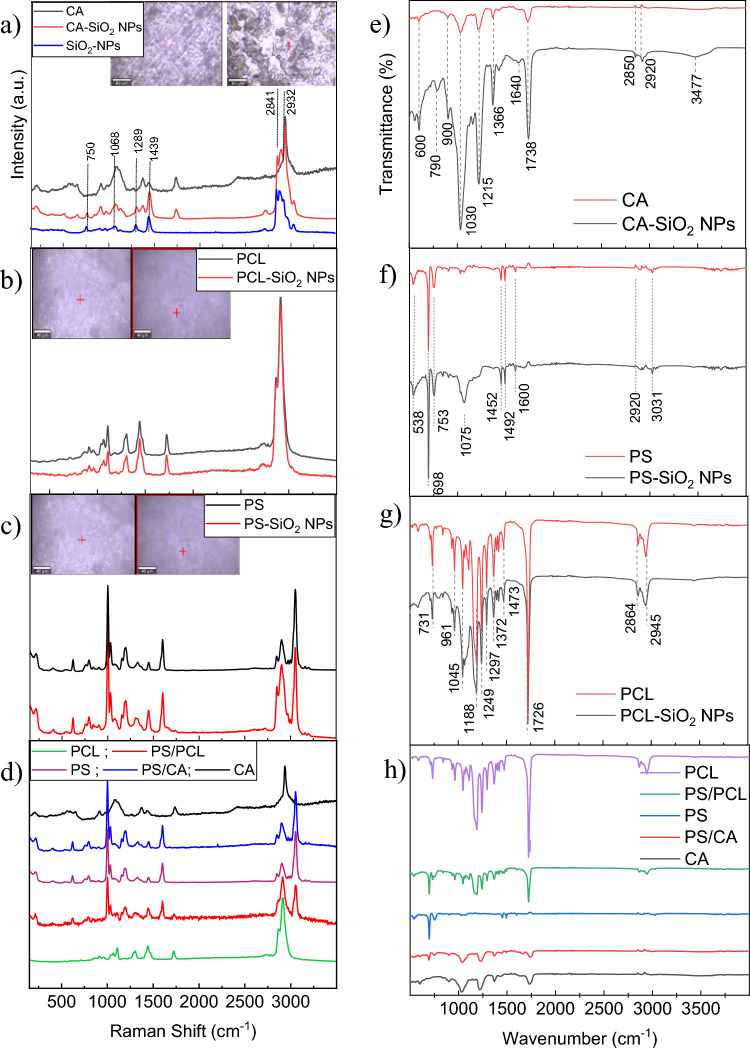
**a**–**c** Raman spectra and **e**–**h** FTIR spectra of the different membranes with and without SiO_2_ NPs.

The Raman spectra of CA membranes display the characteristic cellulose acetate bands: C–H stretching at ~ 2900 cm^−1^, vibrations associated with C–H, O–H, and C–O bonds in the 600–1000 cm^−1^ region, and the C = O vibration of acetyl groups within 1350–1750 cm^−1^. Peaks position and assignments are listed in supplementary Table S1 [22]. Raman spectra of the synthesized SiO_2_ NPs (Fig. 3a) show three transverse optical modes of Si–O-Si: a broad band at 400–600 cm^−1^ (TO1, bending); a band at ~ 800 cm^−1^ (TO2 symmetric stretching), and bands in the 1000–1200 cm^−1^ range (TO3, asymmetric stretching) [23]. In CA-SiO_2_ NPs membranes, the appearance of a feature near ~ 750 cm^−1^ (Si–O-Si bending) supports nanoparticle incorporation. A contribution in the C-H stretching region around ~ 2800 cm^−1^ may indicate residual organics (e.g., trace CTAB), although this assignment is tentative due to overlap with polymer bands [24]. PS membranes, with or without SiO_2_ NPs (Fig. 3b), do not show distinct SiO_2_-related Raman peaks; however, typical PS bands are present, including C–H stretching (2800–2110 cm^−1^), the ring-breathing mode at ~ 1000 cm^−1^, C = C stretching at ~ 1580 cm^−1^, CH_2_ scissoring at ~ 1480 cm^−1^, and C–C stretching at ~ 1155 cm^−1^ (Table S1) [25]. Although SiO_2_ NPs are observed by SEM, PCL membranes with and without SiO_2_ NPs show similar Raman spectra (Fig. 3c), suggesting that the particles are encapsulated within the polymer, more uniformly dispersed, or outside the analyzed region. PCL spectra show the typical bands: C–H stretching (2800–3200 cm^−1^), C = O stretching at ~ 1750 cm^−1^, CH_2_-related modes near ~ 1450 cm^−1^ and ~ 1350 cm^−1^, and C–C/C-COO stretching around ~ 1050 and 950 cm^−1^ [26]. Blended membranes (PS/PCL or PS/CA) show contributions from both polymers; in PS/CA, the dual signatures are less evident, consistent with less uniform composition (Fig. 3d).

FTIR analysis confirmed the presence of cellulose acetate in both membranes, with and without silica nanoparticles. The characteristic CA bands and their vibrational assignments are summarized in Table S2. The spectra indicate that the polymer’s chemical structure is retained following nanoparticle incorporation.

For the CA-SiO_2_ NPs membrane (Fig. 3e), additional absorption bands attributed to silica were observed, namely, a band at around 790 cm^−1^, assigned to the symmetric Si–O-Si vibration, characteristic of amorphous silica [27]. This band is frequently reported as an FTIR fingerprint of the silicon network and complements the evidence obtained by Raman spectroscopy.

Both CA spectra also exhibit a broad band around 3477 cm^−1^, assigned to the O–H stretching of hydrogen-bonded water [27], together with a band near 1640 cm^−1^ corresponding to the bending (scissoring) mode of molecular water [27]. These bands indicate residual moisture, which is commonly reported for hydrophilic or partially hydrophilic polymers such as cellulose acetate, even after drying. Taken together, Raman and FTIR data support silica nanoparticle incorporation while showing no evidence of new chemical bonding or substantial modification of the CA backbone [28].

As shown in Fig. 3f, the FTIR spectra of PS membranes exhibit multiple absorption bands characteristic of PS (Table S2), consistent with the Raman results [29]. In contrast with Raman, FTIR additionally reveals a band at approximately 1075 cm^−1^ [27], assigned to the asymmetric Si–O–Si stretching from silica nanoparticles [27]. The absence of a clear silica Raman signature in PS mats reflects a low local nanoparticles concentration within the probed region, whereas FTIR averages over a larger sampled area.

For the PCL membrane (Fig. 3g), FTIR similarly identifies an additional band around 800 cm⁻^1^, attributed to the symmetric Si–O–Si stretching mode of silica nanoparticles [27, 30], confirming nanoparticle incorporation within the PCL matrix.

The FTIR spectra of the PS/PCL and PS/CA blend membranes (Fig. 3h) confirm the presence of both constituent polymers, with characteristic PS bands observed alongside those of PCL or CA, respectively, in agreement with the spectra of the individual polymers [28–30].

**Figure 4:**
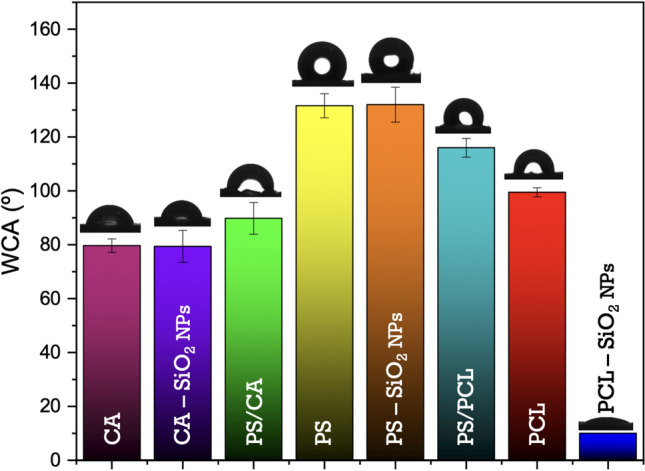
Average water contact angle (WCA) measured for the different membranes produced.

Figure 4 shows the surface interactions with water of different polymeric membranes (CA, PCL, and PS) with or without SiO_2_ nanoparticles, as well as PS/CA and PS/PCL polymer blends, as determined by contact angle measurements. PS and PCL membranes exhibited hydrophobic behavior, with contact angles of approximately 130° and 100°, respectively.

The observed behavior can be attributed, first and foremost, to their intrinsically low polar chemical nature, characterized by low surface energy and a dominant dispersive component [31]. Polystyrene is widely described in the literature as a non-polar polymer, typically exhibiting contact angles with water greater than 90° [32].

Concerning PCL, reported contact angles depend strongly on manufacturing route and surface topography. Smooth PCL films typically show moderate hydrophobicity, whereas fibrous/porous morphologies produced by spinning methods can exhibit higher apparent WCAs due to roughness and air trapping [33, 34]. In blow-spun membranes, the coexistence of fibers, voids, and film-like regions modify the apparent wettability. Here, roughness primarily amplifies the intrinsic wetting tendency of the surface according to Wenzel/Cassie–Baxter descriptions, leading to higher apparent WCAs for hydrophobic compositions.

After incorporation of SiO_2_ NPs into the PCL, the membrane becomes superhydrophilic. The apparent WCA is below the reliable measurement limit because the droplet spreads and/or is absorbed immediately upon contact. Therefore, rather than reporting an exact value (e.g., < 5°), we classify the surface qualitatively as superhydrophilic and report the rapid wetting/absorption behavior. In this instance, silica provides a hydrophilic phase dispersed within a hydrophobic polymer matrix, with surface silanol (Si–OH) groups promoting water affinity [21, 35–37]. It has been demonstrated that these groups exhibit a propensity for interaction with water, thereby increasing the absorption of water molecules and, consequently, reducing the contact angle [35].

Although cellulose acetate has an inherent propensity toward hydrophilicity and polystyrene is highly hydrophobic, the incorporation of silica nanoparticles did not result in substantial alterations in the wettability behavior of these membranes. In the previous studies on cellulose acetate/silica composites, in cases where the silica content is low, the uniform dispersion of nanoparticles within the polymer matrix does not necessarily result in enhanced surface hydrophilicity [38]. Instead, it can be shown that this may even lead to an increase in hydrophobicity, due to the surface chemistry being dominated by the cellulose acetate matrix [38]**.** Therefore, it is likely that silica nanoparticles are embedded within the cellulose acetate matrix or covered by polymer chains. This limits the exposure of silanol groups at the membrane surface and prevents a reduction in water contact angle.

Similarly, in polystyrene membranes, the incorporation of silica did not result in substantial alterations in the contact angle. This finding suggests that the surface chemistry remains predominantly influenced by the polystyrene matrix, thereby constraining the effect of nanoparticles on surface wettability.

As demonstrated in the Supplementary Information (Figures S4), apart from the PCL membrane containing silica nanoparticles, all membranes exhibited stable water contact angles over a period of one minute. This stability suggests that there has been no significant droplet spreading or absorption during the measurement period. This indicates that the surface wettability of the membranes remains essentially unchanged under static conditions.

**Figure 5:**
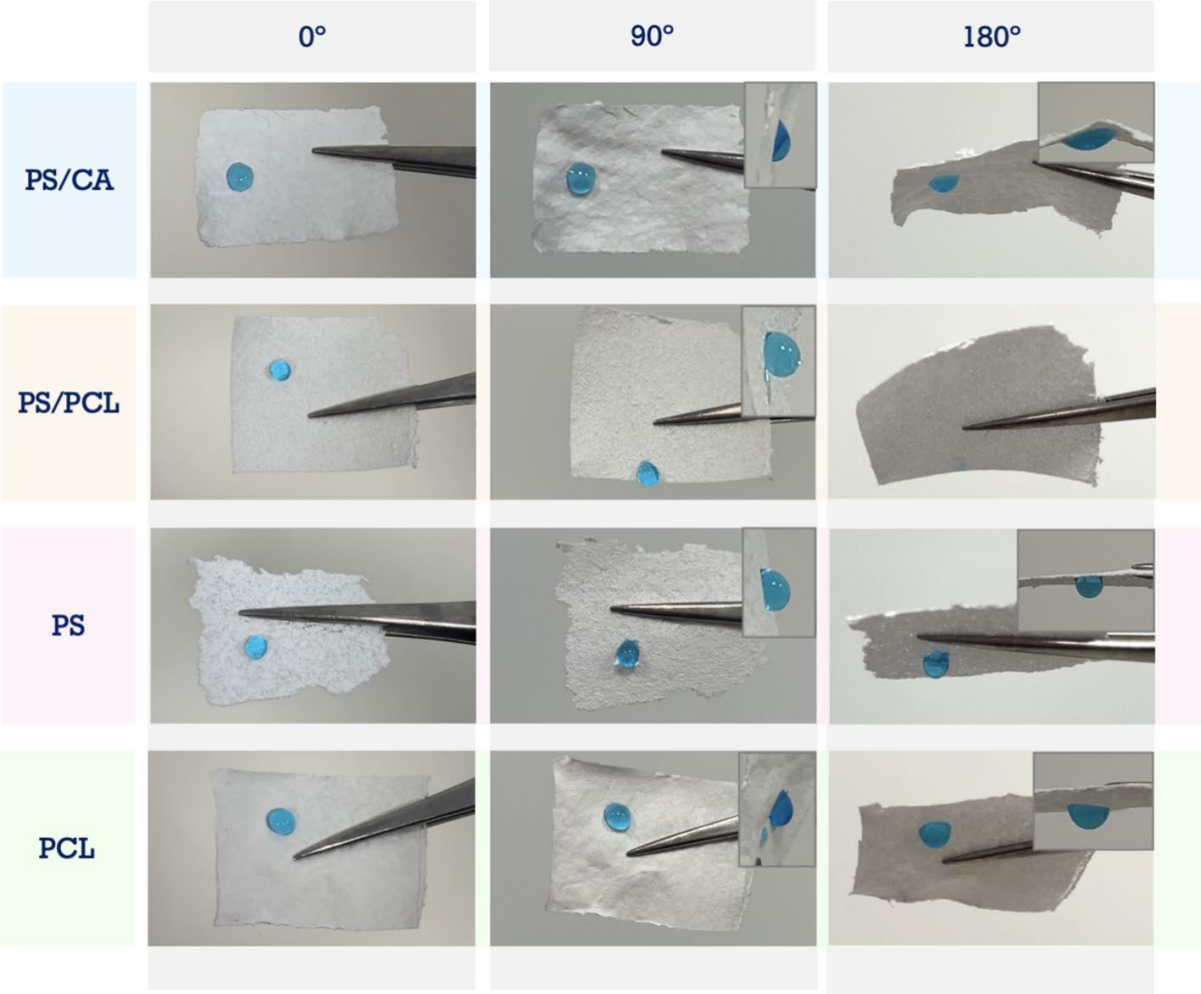
Influence of orientation angle on the hydrophobic behavior of PS/CA, PS/PCL, and PCL membranes: 0° (horizontal plane, droplet on top), 90° (vertical plane), and 180° (horizontal plane, droplet on the underside).

In Fig. 5, PS/CA, PS, and PCL retained water droplets even at high inclination angles, indicating strong interfacial adhesion and drop pinning. Upon rotation to 180° (droplet on the underside), the droplets remained attached, further indicating robust liquid–solid interaction [9]. By contrast, PS/PCL exhibited high water repellence and low droplet adhesion; at low tilt angles, the droplet rapidly slid off, leaving no visible trace of water or dye on the surface [39].

The distinct droplet adhesion observed for membranes with similarly high WCAs can be interpreted using wetting-state concepts often discussed as “lotus” versus “rose-petal” behavior. In a lotus-like state (Cassie–Baxter with low contact angle hysteresis), air pockets and a low real solid–liquid contact fraction yield high droplet mobility and low adhesion, consistent with the PS/PCL membrane where droplets slid at low tilt. In contrast, rose-petal-like behavior corresponds to high apparent WCA but strong pinning (high hysteresis), typically associated with partial liquid penetration into surface asperities (Wenzel or impregnating Cassie states). This explains why PS, PCL, and PS/CA can retain droplets even when inverted: Despite high WCA, the triple line is strongly pinned by the hierarchical roughness and/or partially wetted pores.

**Figure 6:**
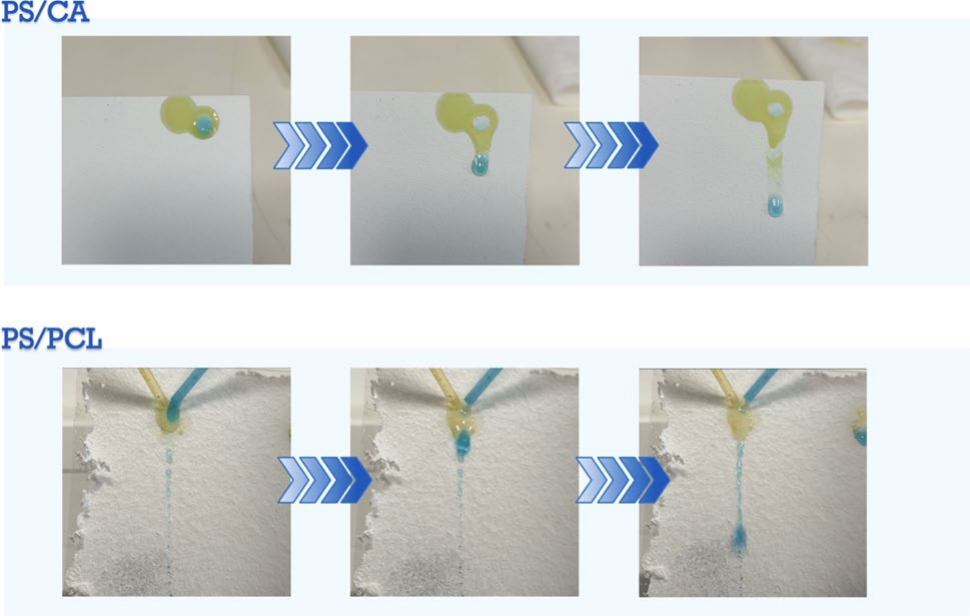
Oil/water separation on PS/CA and PS/PCL membranes at a 90° (vertical) orientation, shown at 0 s, 1 s, and 2 s.

All membranes exhibited oleophilic behavior. On this basis, the hydrophobic blend membranes were evaluated for oil/water separation. Figure 6 shows that the PS/CA and PS/PCL membranes enable selective phase separation driven by contrasting wettability and the different mobility (sliding) of oil and water on the membrane surface.

When a biphasic oil/water droplet was placed on the PS/CA membrane, the oil phase was absorbed, whereas the aqueous phase remained non-wetting. Upon tilting the membrane to a vertical position (90°), the water droplet detached and slid off the surface, allowing collection of the aqueous phase without visible contamination. This behavior reflects the membrane’s higher affinity for oil and its resistance to water wetting, which favors aqueous-phase slippage. The preferential oil uptake is primarily associated with the low surface energy and non-polar character of polystyrene, together with the lower surface tension of oils relative to water, which promotes oil wetting and absorption [31, 40, 41].

The PS/PCL membrane exhibited a faster separation response than PS/CA. On the vertically positioned membrane, the aqueous phase slid off rapidly, consistent with a higher water contact angle (lower water adhesion), while the oil phase was readily absorbed.

Overall, both membranes show promise for oil/water separation. The results further indicate that separation performance depends strongly on membrane composition and surface morphology, which together govern wetting, interfacial adhesion, and slippage of each liquid phase.

In general, oleophilic surfaces with low water adhesion promote selective oil uptake and facilitate drainage of the aqueous phase, whereas surfaces with higher adhesion favors droplet retention even at steep inclination. The tilt tests images support these trends and link the observed droplet dynamics to the physico-chemical and structural characteristics of the membranes.

Quantitatively, PS/CA enabled recovery of 0.39 mL of water from an initial 0.5 mL (78% recovery), whereas the PS/PCL yielded 0.28 mL (56% recovery), using the 45° gravity-driven collection configuration. After settling, no visible oil phase was observed in the collected water in either case, indicating effective oil retention within the limits of visual detection.

Interestingly, despite the lower water recovery, the PS/PCL membrane showed faster apparent transport of the water phase along the surface, consistent with its higher water contact angle and reduced liquid–solid adhesion. By contrast, the PS/CA membrane, with a lower water contact angle (greater water affinity), promoted increased water–surface interaction, which likely contributed to higher recovery but slower droplet motion. These observations emphasize that water recovery is not determined by wettability alone, but by the combined effects of wetting, capillary interactions, and internal membrane structure.

**Figure 7:**
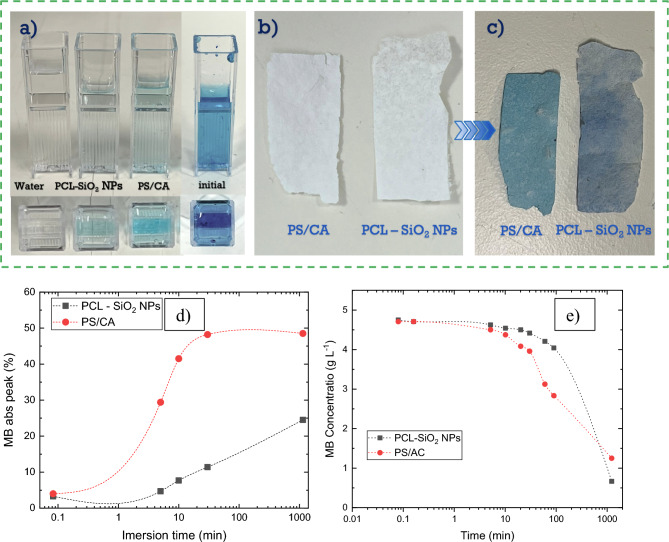
Photographs of the cuvette **a**, membranes before MB immersion **b**, and membranes after 19 h of immersion **c**. Reflectance-derived MB signal at 665 nm as a function of immersion time **d** and MB concentration in solution as a function of cumulative immersion time for the indicated membranes **e**.

To assess the membranes’ capacity to remove dye from water, the interactions between PCL-SiO_2_ NPs and PS/CA membranes with an aqueous methylene blue (MB) solution were investigated. UV–Vis absorbance of the solutions was measured before and after membranes contact at different immersion times and over multiple absorption cycles. In parallel, membrane reflectance spectra were recorded in both the wet and dry states after immersion. Figure 7 a, b and c shows images of the cuvettes and membranes before and after tests.

The MB uptake estimated from membranes reflectance is shown in Fig. 7d, whereas the corresponding MB removal from the aqueous phase is presented in Fig. 7e. The membrane “MB absorbance” derived from reflectance was calculated as the difference between the baseline reflectance and the minimum reflectance at 665 nm, corresponding to the MB absorption maximum (Figure S5).

MB absorption on the PS/CA membrane shows rapid uptake followed by saturation within ~ 30 min, consistent with predominantly surface-limited absorption. For the tested PS/CA sample (area 3.5 cm^2^, thickness 230 μm, and mass 47.5 mg), the maximum removal corresponds to ~ 2.1 mg L^−1^. Given the 1 mL solution volume, this equates to ~ 2.1 μg of MB absorbed, i.e., an apparent uptake of ~ 0.044 mg g^−1^. By contrast, PCL-SiO_2_ NPs membranes exhibit slower uptake at early times but continued absorption over longer immersion periods, consistent with absorption within a porous structure. After 19 h, the solution appears almost colorless (Fig. 7a–c). Notably, for the PCL-SiO_2_ NPs membranes (area 6.9 cm^2^, thickness 118 μm, and mass 24.6 mg), the evolution of the reflectance-derived surface signal does not coincide with the maximum MB removal determined from solution measurements. This discrepancy is likely due to MB uptake occurring within the membrane interior, which is not effectively captured by reflectance (a surface-sensitive measurement), possibly related to a more compact morphology as observed for PS/CA (Fig. 2), which may limit dye penetration relative to the more open/porous PCL-SiO_2_ NPs. Despite this, the overall kinetic trends remain consistent: MB removal is faster for PS/CA because uptake occurs mainly at the surface, whereas internal uptake in PCL-SiO_2_ NPs membranes requires longer contact times. Under the conditions tested (1 mL solution), the PCL-SiO_2_ NPs membranes reduced the MB concentration by ~ 4 mg L^−1^ after 19 h, corresponding to ~ 4 μg removed, i.e., an apparent uptake of ~ 0.16 mg g^−1^ (membrane mass 24.6 mg).

## Conclusions

This work demonstrates blow spinning as a simple, low-cost, and scalable route to fabricate free-standing membranes or coatings from environmentally friendly polymers (PCL and CA), PS, and their blends. Pristine PCL and PS membranes are hydrophobic and oleophilic, while CA membranes are hydrophilic and oleophilic. Incorporation of SiO_2_ nanoparticles renders PCL membranes superhydrophilic while preserving oleophilicity, enabling tuning of wetting behavior via formulation. Oil–water selectivity was demonstrated qualitatively through two-phase droplet tests, where PS/CA and PS/PCL exhibited selective oil uptake with water drainage governed by droplet adhesion (lotus-like vs rose-petal-like behavior). Methylene blue removal tests demonstrated rapid uptake by PS/CA membranes and slower, time-dependent uptake by PCL–SiO_2_ NPs membranes, reflecting distinct dye interaction mechanisms dominated by surface adsorption and internal absorption, respectively. Under the conditions tested, PS/CA exhibited an apparent MB uptake of ~ 0.044 mg g^−1^.

## Supplementary Information

 Below is the link to the electronic supplementary material.

Supplementary file1 (DOCX 1153 KB)
