# Supplementary material for: Influence of silica nanoparticle incorporation on the wettability, oil affinity, and dye removal properties of blow-spun polymeric membranes
Source: J Mater Sci. 2026 Jul 15;61(34):25816–28. doi: 10.1007/s10853-026-13279-6 (PMC13396016; doi:10.1007/s10853-026-13279-6)
Supplement: Supplementary file 1 — Supplementary file1 (DOCX 1153 KB) [file 10853_2026_13279_MOESM1_ESM.docx]

Influence of Silica Nanoparticle Incorporation on the Wettability, Oil Affinity, and Dye Removal Properties of Blow-Spun Polymeric Membranes

A. S. Bento, M. Brito, A. Rovisco, A. C. Baptista, I. Ferreira*

CENIMAT|i3N, Department of Materials Science, School of Science and Technology, NOVA University Lisbon, Caparica, Portugal

*****Corresponding author: imf@fct.unl.pt

**Supplementary Information**

**Experimental details**

The materials used in this work are referred in the following**.**

**Polymers:** Polystyrene (PS, Mw ~350 kDa), Cellulose acetate (CA, Mn ~50,000), Polycaprolactone (PCL, Mn~80,000) and Poly(vinyl alcohol) (PVA, Mw 89,000-98,000, 99+% hydrolyzed) were all purchased from Sigma-Aldrich.

**Solvents:** Dichloromethane (DCM, 99.8%), N,N-Dimethylformamide (DMF, 99.8), Acetone (99.8%) and N,N-Dimethylacetamide (DMAC, 99.8) were all purchased from Carlo Erba Reagents.

**Silica nanoparticles synthesis**: Tetraethyl orthosilicate (TEOS, 98%, Sigma-Aldrich), Hexadecyltrimethylammonium bromide (CTAB, **≥ 98%, Sigma-Aldrich), Sodium Hydroxide (NaOH 98%, Labchem).**

**Methylene blue (MB),** Mw=319.85 g/mol, Thermo Scientific Chemicals.

**Results**

The reflectance of the membranes was measured after immersion in a methylene blue (MB) solution and subsequent drying in an oven at 50 °C during 5 min. The absorbance of Millipore (MP) water and MB solutions was first measured to generate a calibration curve. The resulting absorbance spectra are shown in Figure S1A, and the corresponding calibration curve was obtained using the absorbance value at main peak (665 nm) for each concentration (Figure S1 B).

B

A

Figure S1: (A) Absorbance spectra of Millipore (MP) water and methylene blue (MB) solutions at different concentration; and (B) Corresponding calibration curve of absorbance versus MB concentration.

The absorbance spectrum of the solution was measured after immersion of 1 cm x 3 cm samples of PCL-SiO_2_ NPs (Figure S2 A) and PS/CA (Figure S2 B) membranes. The absorbance was cumulative, meaning that the same solution was measure after consecutive immersions of sample 1 for 5 s (5s-1), sample 2 for 5 s (5s-2), and so forth.

Av

B

Figure S2: Absorbance spectra of Millipore (MP) water and methylene blue (MB) solution after consecutive immersions of sample1 (denoted as “1” in the legend) and sample 2 (denoted as “2” in the legend) for each polymeric membrane: PCL-SiO_2_ NPs (A) and PS/CA (B).

The absorbance peak of MB was plotted for the different membranes as a function of cumulative immersion time for samples 1 and 2 (Figure S3). Immersion time was considered cumulative, meaning that the immersion of one sample removes a quantity of MB from the solution, and the immersion of the second sample for an equivalent period is treated as the equivalent of double the immersion time of a single sample. For example, the final immersion period was 19 h (1140 min), but the total cumulative immersion time, including all previous sample immersion was 1230.16 min.

Figure S3 – Absorbance the MB peak in the solution as a function of cumulative immersion time for the different membranes.

For the PCL membranes (Figure S4), the reflectance in the wet state is systematically lower than that in the dry state. This behaviour is consistent with the presence of water retained within the pores and on the membrane surface, which reduces diffuse reflection by increasing light absorption phenomena. Upon drying, the water evaporates, restoring the air–polymer interfaces responsible for internal scattering and, consequently, for the higher reflectance observed [1,2]. In addition, the presence of water is confirmed by bands in the NIR (~1400 and 1900 nm), attributed to combinations of O–H vibrations, which decrease after membrane drying [3].

When analysing the peaks in the visible range, a band at ~400–500 nm can be identified, attributed to electronic transitions of the dye adsorbed by the membrane [4]. The intensity of this band increases with contact time, indicating greater dye accumulation in the PCL membrane and reflecting the progressive transfer of dye from solution to the polymer matrix.

The evolution of the wettability measurement in membranes during 1 min is shown in Figure S4 from a) to e). In the period considered the water droplet is constant or slightly changes. WCA values were recorded immediately after droplet deposition and monitored over time to assess surface wettability and water/surface interaction stability.

| 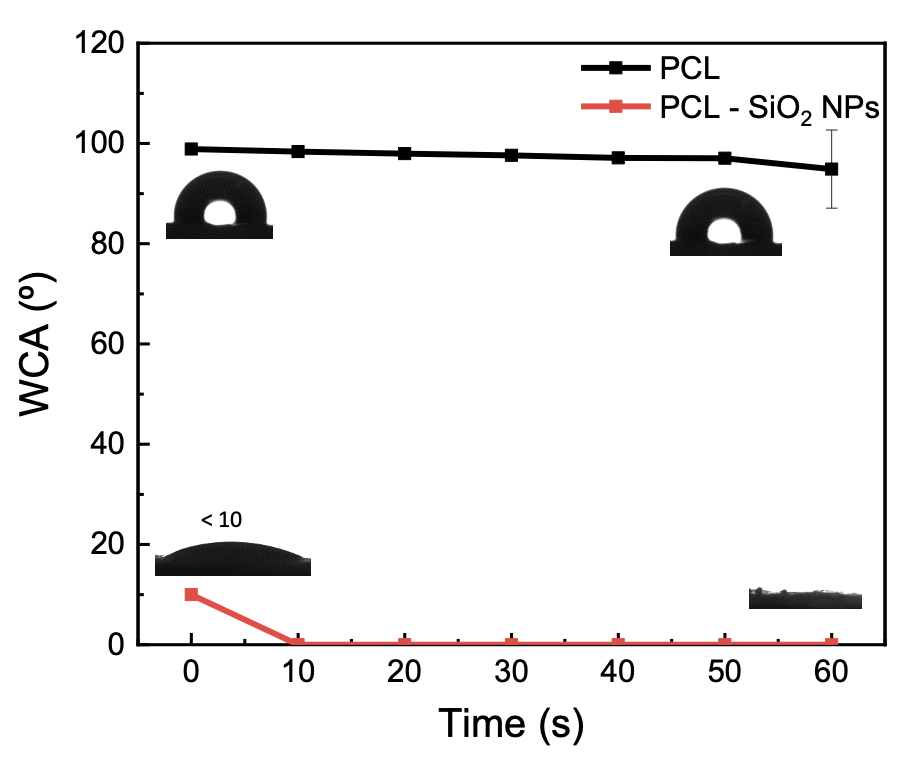  a) | 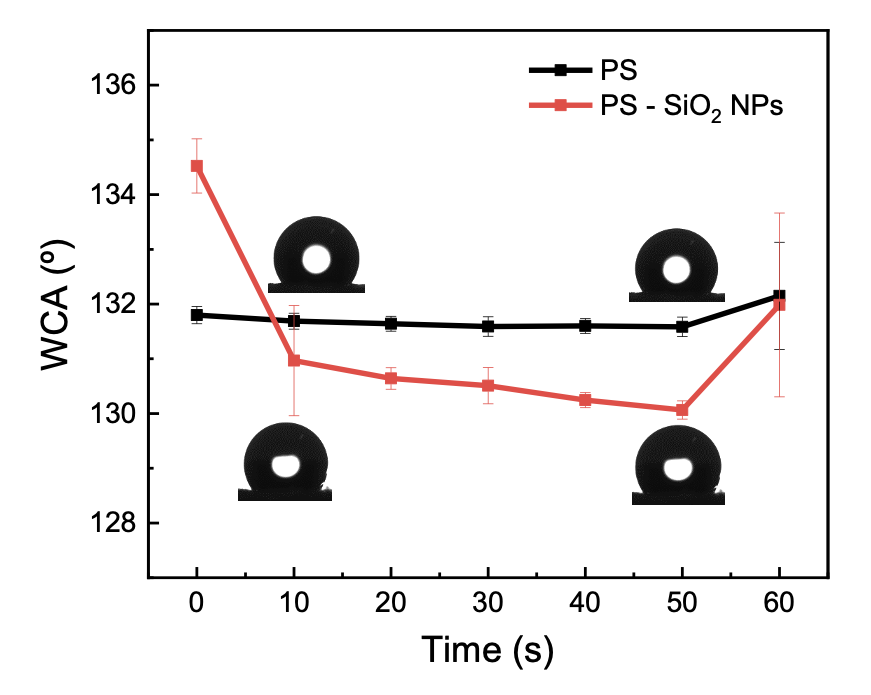  b) |
| --- | --- |
| 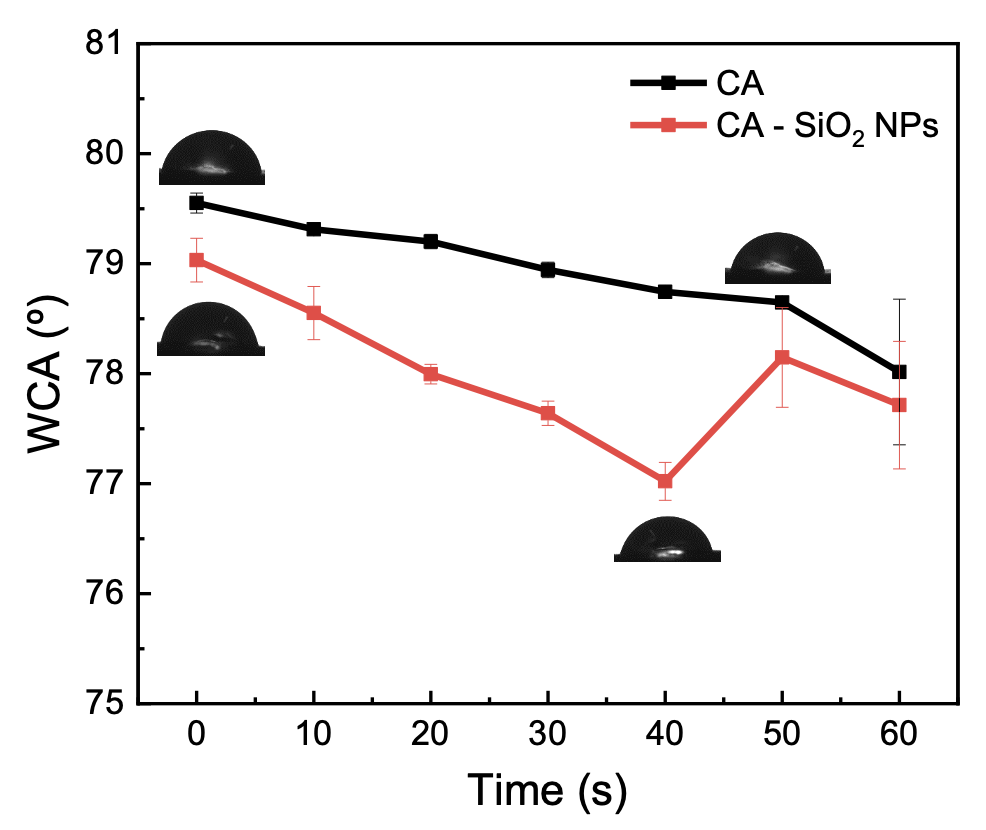  c) | 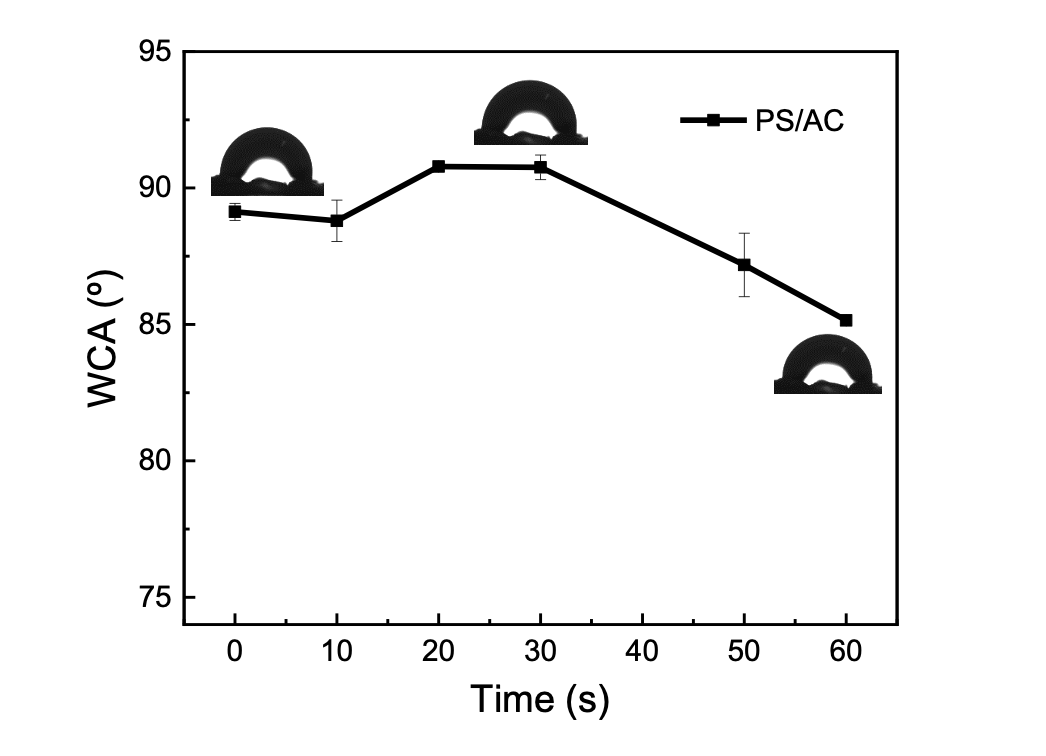  d) |
| 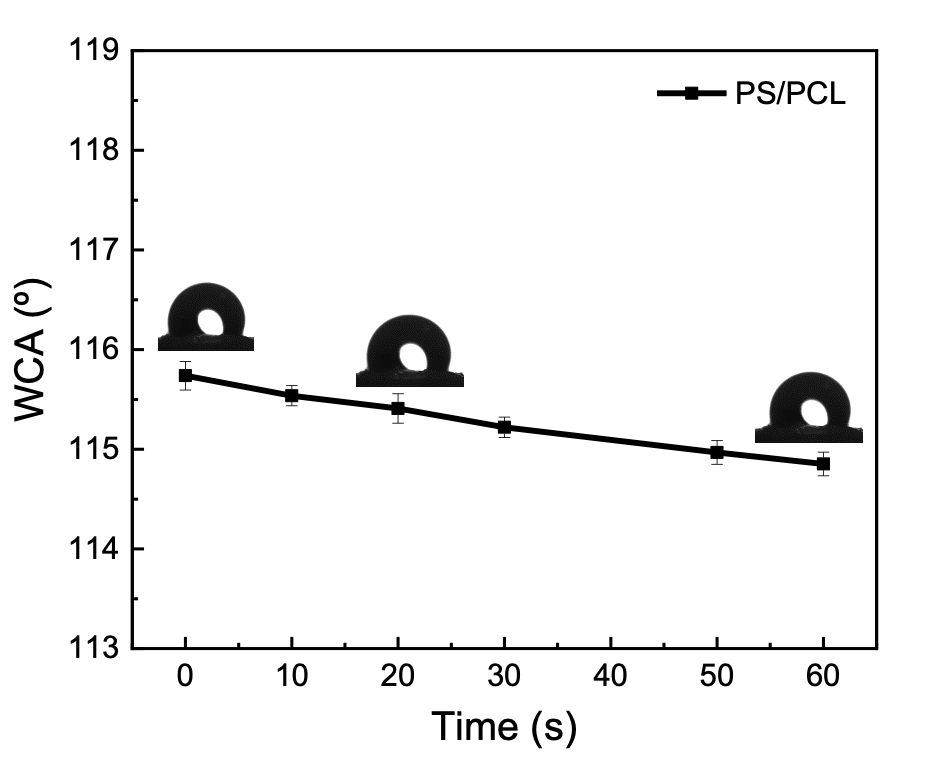  e) |  |

Figure S4 – Water contact angle (º) as a function of time for: a) PCL and PCL with SiO₂, b) CA and CA with SiO₂ NPs, c) PS and PS with SiO₂ NPs, d) PS/CA, and e) PS/PCL polymeric surfaces.

The reflectance spectra of the membranes after immersion in the MB solution were measured both in wet state and after drying it for 5 min in an oven at 50 °C. Representative spectra for one sample are shown in Figure S5A for PCL-SiO_2_ NPs membranes and Figure S5B for PS/CA membranes. Figure S5C compares the reflectance of both membranes in the wet and dry states.

b)

a)

c)

Figure S5: Reflectance spectra of sample 1 for membranes of a) PCL-SiO_2_ NPs, b) PS/CA; and c) comparison of wet and dry states for the same immersion time.

**Raman peaks**

Table S1 - Main Raman peaks obtained and corresponding modes from literature for the polymers studied.

| **Cellulose Acetate Raman (cm^-1^) [5]** | | | **0btained peaks (cm^-1^)** |
| --- | --- | --- | --- |
| 2934 cm^-1^ | C-H | Stretching | 2840-2939 |
| 1121 | C-O-C | Asymmetric stretching vibration of glycosidic linkage | 1128 |
| 1081 | C-OH | Pyranose ring signal | 1078 |
| 1265 | C-OH | Bonds present in the rings | 1270 |
| 1736 | C=O | Vibration of the carbonyl group from Acetyl group | 1736 |
| 1435 |  |  | 1438 |
| 1382 |  |  | 1369 |
| 978 | C-O | Symmetric and asymmetric vibrations of the C-H bond in the acetyl groups | 978 |
| 906 | C-H |  | 910 |
| 834 | O-H |  |  |
| 659 | C-OH |  |  |
| **Polycaprolactone [6]** | | |  |
| 685-1018 | C-COO | stretch | 960,916, 867 |
| 997-1150 | C-C |  | 1090-1066 |
| 1200-1362 | CH_2_ | twist | 1301 |
| 1362-1540 | CH_2_ | blend | 1438 |
| 1600-1850 | C¼O | stretch | 1723 |
| 2800-3200 | C-H | stretch | 2858-2907 |
| **Polystyrene [7]** | | |  |
| 621 |  | Ring deformation mode | 621 |
| 795 | C-H | Out-of-plane deformation | 795 |
| 1001 |  | Ring breathing mode | 997 |
| 1031 | C-H | In-plane deformation | 1030 |
| 1155 | C-C | Stretch | 1198 |
| 1450 | CH2 | Scissoring | 1445 |
| 1583 | C=C | Stretch |  |
| 1602 |  | Ring skeletal stretch | 1600 |
| 3000-3100 | C-H | Stretch | 3049, 2900, 2841 |
| **Silica [8]** | | | |
| 1065 | Si–O–Si | asymmetric vibrational mode | 1068 (in CA) |
| 750-850 | Si-O-Si | bending vibrations |  |
| 472 | Si—O—Si | Symmetric stretching |  |

**FTIR peaks**

Table S2 - Main FTIR peaks obtained and corresponding modes from literature for the polymers studied.

| **Cellulose Acetate Raman Peaks (cm^-1^) [9]** | | | **0btained peaks (cm^-1^)** |
| --- | --- | --- | --- |
| 2930 | C-H | asymmetric stretching of the CH_3_ group | 2920 |
| 2860 | C-H | asymmetric stretching of the methylene group | 2850 |
| 1742 | C=O | stretching vibration modes of the C=O bond of the ester group in the polymer |  |
| 1431.18 | CH_2_ | Deformation vibration |  |
| 1370 | C–H | symmetric bending | 1366 |
| 1229 | C-O | C–O asymmetric stretching of the carboxy- late group | 1215 |
| 1043 | C-O-C | C–O–C asymmetric stretching of ether group in the pyranose ring | 1030 |
| 904.61 | C-H | C–H out of plane deformation | 900 |
| 600 | OH | OH out of plane deformation | 600 |
| **Polycaprolactone [10] [11]** | | |  |
| 2940,2863 | CH | stretch | 2945,2864 |
| 1726 | C=O | stretching | 1726 |
| 1471 | CH_2_ | antisymmetric bending | 1473 |
| 1419 | O-CH_2_ | bending |  |
| 1366 | CH_2_ | symmetric bending | 1372 |
| 1294,1240,1186 | C-O-C | stretch | 1297,1249,1188 |
| 1045 |  | C–O stretching and C–H bending | 1045 |
| 961 | C-C | skeletal C-C and C-H wagging | 961 |
| 733 | C–H | C-H Bending | 731 |
| **Polystyrene [12]** | | |  |
| 3446.5 | O-H | stretching vibration |  |
| 3060.8, 3026 | C-H | aromatic C-H stretching vibration absorption | 3031 |
| 2921.9, 2846.6 | C-H | existence of methylene | 2920 |
| 1600.8,1492.7,1452.5 | C=C | aromatic C=C stretching vibration absorption | 1600,1492,1452 |
| 1027.9 | C-H | Stretching vibration |  |
| 756, 698.2 | C-H | out-of-plane bending vibration absorption | 753,698 |
| 538.1 | C-H | out-of-plane bending vibrations | 538 |
| **Silica [13]** | | | |
| 1090 | Si-O | asymmetric vibration | 1075 (in PS) |
| 950 | Si-OH | asymmetric vibration |  |
| 793 | Si-O | Symmetric vibration | 790 (in CA) |

**Supplementary information**

**References**

1. Zhu C, Kanaya Y (2023) Eliminating the interference of water for direct sensing of submerged plastics using hyperspectral near-infrared imager. Sci Rep 13:15991. https://doi.org/10.1038/s41598-023-39754-7

2. Nielsen KH, Kittel T, Wondraczek K, Wondraczek L (2014) Optical breathing of nano-porous antireflective coatings through adsorption and desorption of water. Sci Rep 4:6595. https://doi.org/10.1038/srep06595

3. Torres LF, Oliveira MR, Barbalho TCS, et al (2025) The use of NIR spectroscopy for the quantification of water content and compositional analysis in compressed gas-systems. Vibrational Spectroscopy 139:103815. https://doi.org/10.1016/j.vibspec.2025.103815

4. Fernández-Pérez A, Marbán G (2020) Visible Light Spectroscopic Analysis of Methylene Blue in Water; What Comes after Dimer? ACS Omega 5:29801–29815. https://doi.org/10.1021/acsomega.0c03830

5. Sánchez-Márquez JA, Fuentes-Ramírez R, Cano-Rodríguez I, et al (2015) Membrane Made of Cellulose Acetate with Polyacrylic Acid Reinforced with Carbon Nanotubes and Its Applicability for Chromium Removal. International Journal of Polymer Science 2015:1–12. https://doi.org/10.1155/2015/320631

6. Baranowska-Korczyc A, Warowicka A, Jasiurkowska-Delaporte M, et al (2016) Antimicrobial electrospun poly(ε-caprolactone) scaffolds for gingival fibroblast growth. RSC Adv 6:19647–19656. https://doi.org/10.1039/C6RA02486F

7. Menezes DB, Reyer A, Marletta A, Musso M (2017) Glass transition of polystyrene (PS) studied by Raman spectroscopic investigation of its phenyl functional groups. Mater Res Express 4:015303. https://doi.org/10.1088/2053-1591/4/1/015303

8. Cassetta M, Sorarù GD, Callone E, et al (2025) From SiO1.5CH3 to vitreous SiO2: A structural evolution study. Journal of Non-Crystalline Solids 648:123327. https://doi.org/10.1016/j.jnoncrysol.2024.123327

9. Santos-Sauceda I, Castillo-Ortega MM, Del Castillo-Castro T, et al (2021) Electrospun cellulose acetate fibers for the photodecolorization of methylene blue solutions under natural sunlight. Polym Bull 78:4419–4438. https://doi.org/10.1007/s00289-020-03324-y

10. Zeng W, Cheng N, Liang X, et al (2022) Electrospun polycaprolactone nanofibrous membranes loaded with baicalin for antibacterial wound dressing. Sci Rep 12:10900. https://doi.org/10.1038/s41598-022-13141-0

11. Peter A, Monea MB, Mihaly Cozmuta A, et al (2025) Polylactic Acid-Polycaprolactone Scaffolds Prepared by Porogen Leachin, J Environ Anal Toxicol 2015, 5:6. https://doi.org/ 10.4172/2161-0525.1000336

12. Adeniyi AG, Abdulkareem SA, Emenike EC, et al (2024) Mechanical and chemical characterization of biochar-reinforced polystyrene composites. BMC Chemistry 18:246. https://doi.org/10.1186/s13065-024-01365-2

13. Bm V, M V (2015) Photocatalytic Degradation of Toxic Methyl Red Dye Using Silica Nanoparticles Synthesized from Rice Husk Ash. J Environ Anal Toxicol 05: https://doi.org/10.4172/2161-0525.1000336
